# Cell entry mechanisms of porcine enteric coronaviruses

**DOI:** 10.1016/j.jbc.2026.111250

**Published:** 2026-02-05

**Authors:** Yiping Wang, Fei Zhao, Qin Zhao, Senyan Du, Yiping Wen, Rui Wu, Sanjie Cao, Feng Cong, Xiaobo Huang

**Affiliations:** 1 Department of Preventive Veterinary Medicine, Research Center for Swine Diseases, College of Veterinary Medicine, Sichuan Agricultural University, Chengdu, China; 2 Agricultural Animal Diseases and Veterinary Public Health Key Laboratory of Sichuan Province, Sichuan Agricultural University, Chengdu, China; 3 Engineering Research Center of Southwest Animal Disease Prevention and Control Technology of Ministry of Education, Sichuan Agricultural University, Chengdu, China; 4 Key Laboratory of Agricultural Bioinformatics of Ministry of Education, Sichuan Agricultural University, Chengdu, China; 5 College of Animal Science & Technology, Zhongkai University of Agriculture and Engineering, Guangzhou, China

**Keywords:** porcine enteric coronaviruses, TGEV, PEDV, SADS-CoV, PDCoV, virus entry

## Abstract

Porcine enteric coronaviruses, including transmissible gastroenteritis virus (TGEV), porcine epidemic diarrhea virus (PEDV), swine acute diarrhea syndrome coronavirus (SADS-CoV), and porcine deltacoronavirus (PDCoV), cause severe watery diarrhea, vomiting, dehydration, and high mortality in piglets, leading to enormous economic losses in the swine industry worldwide. They have the capability to infect a variety of cell lines from pigs, humans, and other animals, with high risks of interspecies transmission and potential threats to public health. These viruses employ their spike glycoproteins to engage with various receptors, coreceptors, cofactors, and other host factors that further mediate membrane fusion to accomplish the entry process. This review summarizes the recent findings regarding the pathways, receptors, coreceptors, cofactors, and other host factors utilized by TGEV, PEDV, SADS-CoV, and PDCoV for cellular entry. Several important targets for antiviral therapeutics and some key aspects of the entry process for these viruses that await discovery are highlighted. A comprehensive understanding of the entry mechanisms of porcine enteric coronaviruses will provide new insight into the development of novel antiviral therapeutic strategies.

Coronaviruses are a large group of enveloped positive-sense single-stranded RNA viruses that belong to the Coronaviridae family of the *Nidovirales* order (1). They are divided into four genera: *Alphacoronavirus*, *Betacoronavirus*, *Gammacoronavirus*, and *Deltacoronavirus* (1). Currently, the known porcine enteric coronaviruses that cause gastrointestinal infections in pigs include three alphacoronaviruses transmissible gastroenteritis virus (TGEV), porcine epidemic diarrhea virus (PEDV), and swine acute diarrhea syndrome coronavirus (SADS-CoV), as well as one deltacoronavirus porcine deltacoronavirus (PDCoV) (2, 3). TGEV was first identified in 1946 and has been circulating in pig populations for decades (2, 3). PEDV, PDCoV, and SADS-CoV were identified in 1971 (reemerged in 2010), 2014, and 2017, respectively, and are recognized as emerging and reemerging swine coronaviruses, with PEDV, SADS-CoV, and PDCoV presumably originated from bat or sparrow coronavirus, respectively (2, 3). Infections of these viruses in pigs result in severe digestive tract related clinical symptoms, including watery diarrhea, vomiting, dehydration, and high mortality, leading to enormous economic losses in the swine industry worldwide (2, 3). In addition to their natural host pigs, these porcine enteropathogenic coronaviruses have the capability to infect a large variety of cell lines from humans and other animals, thus holding potential risks of cross-species transmission (4, 5, 6, 7).

The basic genomic organization is shared among swine enteric coronaviruses, with the genome size of approximately 25 to 28 kilobases (2, 3). Each genome consists of a 5′-untranslated region (UTR), open reading frame 1a (ORF1a), ORF1b, the structural protein genes encoding spike (S), envelope (E), membrane (M), and nucleocapsid (N) proteins, the accessory genes encoding one or more accessory proteins (ORF3a, ORF3b, and ORF7 for TGEV; ORF3 for PEDV; NS3a, NS7a, and NS7b for SADS-CoV; and NS6, NS7, and NS7a for PDCoV), and a 3′-UTR (2, 3). ORF1a and ORF1b encode polyprotein 1a (pp1a) and pp1b, respectively, which are further cleaved to generate a panel of nonstructural proteins involved in viral replication and transcription. Among the structural proteins, the S glycoprotein mediates viral entry, the E protein forms an ion channel in the viral membrane and is involved in viral assembly, the M protein is required for incorporating key viral components into new viral particles during morphogenesis, and the N protein participates in virion packaging by binding to viral genome (8). The S glycoprotein exists as a homotrimer, with each monomer comprised of two domains, S1 and S2. The S1 subunit contains the receptor-binding domain (RBD) responsible for engagement with the host receptors at the cell surface, while the S2 subunit mediates subsequent membrane fusion to allow the virus to enter the cytoplasm. Aside from its essential role in viral entry, the S glycoprotein is the primary target for neutralizing antibodies against coronaviruses.

Understanding of the entry mechanisms of porcine enteric coronaviruses is critically important for developing new and effective antiviral therapeutic strategies. Consistent with this, neutralizing antibodies that block the interaction between PDCoV and its host receptors have been demonstrated to be effective in repressing viral infection (9, 10, 11). In this review, we summarize the recent findings concerning the pathways, receptors, coreceptors, cofactors, and other host factors used by TGEV, PEDV, SADS-CoV, and PDCoV for cellular entry. We provide insight into the entry factors that may be amenable to antiviral targeting and highlight the key aspects of the entry process that await discovery. A comprehensive understanding of the entry mechanisms of porcine enteric coronaviruses will contribute to the development of novel antiviral therapeutic strategies.

## Entry pathways of porcine enteric coronaviruses

As obligate intracellular parasites, viruses must first enter host cells such that to cause infection successfully. Virus entry is a complicated multistep process that begins with binding to cell-surface receptors and terminates with the delivery of the viral genome into the cytoplasm. To overcome physical barriers of cells, viruses typically utilize two main strategies to enter cells: the endocytic and nonendocytic routes (12). The endocytic route of virus entry is usually comprised of clathrin-mediated endocytosis (CME), caveolae-mediated endocytosis (CavME), and macropinocytosis (12, 13). The nonendocytic route of virus entry involves direct fusion of viral membrane with the plasma membrane at the cell surface in a pH-independent manner (12). Viruses can enter cells by making use of either endocytic or nonendocytic pathways, or even both. The entry pathways by which TGEV, PEDV, SADS-CoV, and PDCoV enter host cells are summarized in Table 1.

**Table 1:** The entry pathways used by TGEV, PEDV, SADS-CoV, and PDCoV

Virus	Cell type	Direct fusion	Endocytosis	Clathrin	Dynamin	Caveolae	Cholesterol	Macropinocytosis	Small GTPases	Low pH	References
TGEV	MDCK expressing pAPN	ND	✓	ND	ND	ND	ND	ND	ND	✓	(15)
	ST	ND	✓	✓	✓	✓	✓	ND	ND	✓	(15, 16, 17, 18)
	IPEC-J2	ND	✓	✓	ND	✓	✓	ND	ND	ND	(19)
PEDV	Vero	✓	✓	✓	✓	✓	✓	ND	Rab5/7	✓	(20, 21, 22, 23, 25)
	IPEC-J2	ND	✓	✓	✓	✓	✓	ND	ND	✓	(21)
	PK15, IPI-2I, HuH7	ND	✓	ND	ND	ND	ND	ND	ND	✓	(24)
SADS-CoV	Vero	✓	✓	✓	✓	✓	✓	✓	Rab5/7/9	✓	(26, 27)
	IPI-2I	ND	✓	✓	✓	✓	✓	✓	Rab5/7/9	✓	(26)
	HuH7, HuH7.5.1, HeLa	✓	ND	ND	ND	ND	ND	ND	ND	ND	(28, 29)
PDCoV	ST	✓	✓	✓	✓	✓	✓	✗	✗	✓	(30, 31, 32, 34, 35)
	IPI-2I	✓	✓	✓	✓	✗	ND	✓	Rab5/7	✓	(30, 31)
	HuH7	✓	✓	ND	ND	ND	ND	ND	ND	ND	(33)
	LLC-PK1	ND	✓	✗	ND	✓	ND	✗	ND	✓	(31, 35, 36)
	PK15	ND	✓	✗	✓	✓	✓	✗	ND	✗	(37)
	DF-1	ND	✓	✓	✓	✗	ND	✗	Rab5/7	✓	(38)

DF-1, Douglas Foster-1, immortalized chicken embryo fibroblasts; HeLa, human cervical carcinoma cells; HuH7, human hepatoma cells; HuH7.5.1, a subclone of the parent HuH7 cells; IPEC-J2, intestinal porcine epithelial cells-jejunum 2; IPI-2I, porcine ileum epithelial cells; LLC-PK1, porcine kidney cells; MDCK, Madin-Darby canine kidney cells; ND, not determined; PK15, porcine kidney cells; Vero, African green monkey kidney cells.

**Figure 1:**
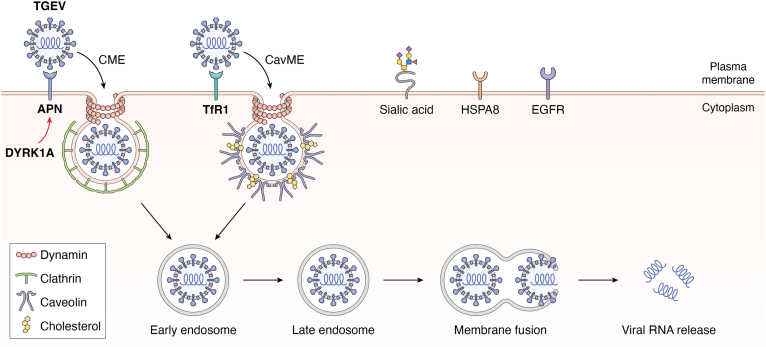
**Schematic diagram of TGEV entry pathways**. Multiple cell-surface molecules are involved in the entry of TGEV, including APN, TfR1, sialic acid, HSPA8, and EGFR (summarized in Table 2). Following attachment to target cells, TGEV virions are internalized through clathrin-mediated endocytosis (CME) and caveolae-mediated endocytosis (CavME) in a dynamin- and cholesterol-dependent manner, and transported to early endosome, then to late endosome, where membrane fusion takes place and viral RNA is released to the cytoplasm. DYRK1A promotes TGEV attachment and internalization by increasing APN expression. APN, aminopeptidase N; DYRK1A, dual-specificity tyrosine phosphorylation-regulated kinase 1A; EGFR, epidermal growth factor receptor; HSPA8, heat shock protein family A member 8; TfR1, transferrin receptor 1; TGEV, transmissible gastroenteritis virus.

Early studies on revealing the entry pathways of TGEV using Madin-Darby canine kidney (MDCK) cells expressing recombinant porcine aminopeptidase N (pAPN), the major receptor for TGEV (14), demonstrated that TGEV entry into cells is through receptor-mediated endocytosis and requires an acidified intracellular environment (15). In the subsequent studies using swine testis (ST) cells, TGEV was demonstrated to be internalized into cells *via* CME and CavME, which was dependent on actin, dynamin 2, cholesterol, and low pH (15, 16, 17, 18) (Fig. 1, which summarizes the pathways, receptors, coreceptors, and other host factors used by TGEV for cellular entry). The same endocytic routes were also used by TGEV to enter intestinal porcine epithelial cells-jejunum 2 (IPEC-J2) (19). However, the detailed trafficking process of TGEV virions inside the cells has not been determined.

**Figure 2:**
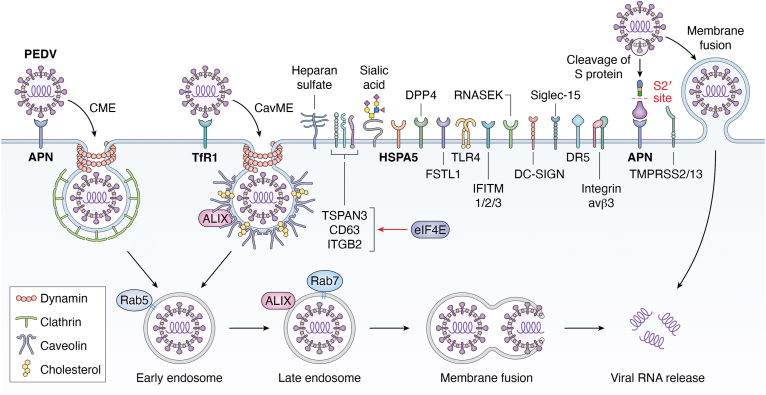
**Schematic diagram of PEDV entry pathways**. Multiple cell-surface molecules participate in the entry of PEDV, including APN, TfR1, heparan sulfate, sialic acid, HSPA5, DPP4, FSTL1, TLR4, IFITM1/2/3, RNASEK, DC-SIGN, Siglec-15, DR5, integrin avβ3, and TMPRSS2/13 (summarized in Tables 2 and 3). Upon binding to target cells, PEDV viral particles are internalized through clathrin-mediated endocytosis (CME) and caveolae-mediated endocytosis (CavME) in a dynamin- and cholesterol-dependent fashion. Trafficking of PEDV virions from early endosome to late endosome requires the small GTPases Rab5 and Rab7, as well as the ESCRT-I accessory protein ALIX. Viral RNA is released to the cytoplasm after membrane fusion. Notably, PEDV virions can enter target cells through membrane fusion at the cell surface by TMPRSS2/13-mediated cleavage of S protein. Besides, eIF4E promotes PEDV attachment by enhancing the expression of membrane-residential molecules TSPAN3, CD63, and ITGB2. ALIX, ALG-2-interacting protein X; APN, aminopeptidase N; DC-SIGN, dendritic cell-specific intercellular adhesion molecule-3-grabbing nonintegrin; DPP4, dipeptidyl peptidase 4; DR5, death receptor 5; eIF4E, eukaryotic initiation factor 4E; ESCRT, endosomal sorting complexes required for transport; FSTL1, follistatin-like 1; HSPA5, heat shock protein family A member 5; IFITM, interferon-induced transmembrane protein; ITGB2, integrin subunit beta 2; PEDV, porcine epidemic diarrhea virus; RNASEK, ribonuclease kappa; Siglec, sialic acid-binding immunoglobulin-type lectin; TfR1, transferrin receptor 1; TLR4, toll-like receptor 4; TMPRSS, transmembrane protease serine; TSPAN3, tetraspanin 3.

Through single-virus tracking technology using lipophilic 1,1′-dioctadecyl-3,3,3′,3′-tetramethylindodicarbocyanine (DiD)-labeled PEDV, both CME and CavME were demonstrated to be involved in the entry of PEDV into Vero cells (20) (Fig. 2, which summarizes the pathways, receptors, coreceptors, cofactors, and other host factors used by PEDV for cellular entry). The process of PEDV penetrating cell membrane is very swift, with an average time of only 3 min (20). Following internalization into cells, PEDV is trafficked from clathrin- and caveolae-structures to early endosome, and then to late endosome, where viral fusion takes place (20). The completion of entire endocytosis process of PEDV into Vero cells requires dynamin 2, cholesterol, and the small GTPases Rab5 and Rab7, and is dependent on low pH (20, 21, 22, 23). Furthermore, PEDV also relies on receptor-mediated endocytosis and low pH to enter porcine kidney PK15 cells, porcine intestinal epithelial IPEC-J2 cells, porcine ileum epithelial IPI-2I cells, and human hepatoma HuH7 cells (21, 24). In addition, PEDV can enter cells through S protein-mediated fusion at the plasma membrane (25) (Fig. 2).

**Figure 3:**
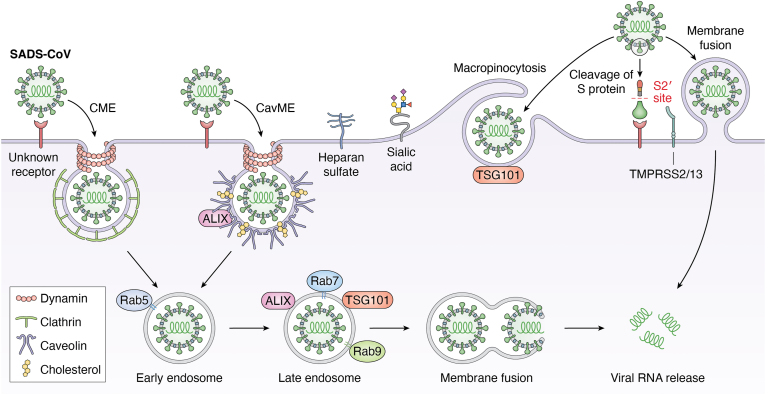
**Schematic diagram of SADS-CoV entry pathways.** Although several cell-surface molecules involved in SADS-CoV entry have been identified, including heparan sulfate, sialic acid, and TMPRSS2/13 (summarized in Table 2), the specific functional receptors for SADS-CoV entry are still unknown. After engagement with the unknown receptors on target cells, SADS-CoV virions are internalized through clathrin-mediated endocytosis (CME), caveolae-mediated endocytosis (CavME), and macropinocytosis. SADS-CoV viral particles are trafficked from early endosome to late endosome, where membrane fusion occurs prior to releasing of viral RNA to the cytoplasm. The entry process of SADS-CoV depends on dynamin and cholesterol, and requires the small GTPases Rab5, Rab7, and Rab9, as well as the ESCRT-I subunit TSG101 and its accessory protein ALIX. In addition, SADS-CoV viral particles can enter target cells through viral fusion at the plasma membrane *via* TMPRSS2/13-mediated cleavage of S protein. SADS-CoV, swine acute diarrhea syndrome coronavirus; ESCRT, endosomal sorting complexes required for transport; TSG101, tumor susceptibility gene 101; ALIX, ALG-2-interacting protein X; TMPRSS, transmembrane protease serine.

SADS-CoV can use both endocytic and nonendocytic (direct fusion) routes to enter target cells, including Vero and HuH7 cells (26, 27, 28, 29) (Fig. 3, which summarizes the pathways, receptors, cofactors, and other host factors used by SADS-CoV for cellular entry). It employs three endocytic pathways of CME, CavME, and macropinocytosis to enter Vero and IPI-2I cells (26). The endocytosis process requires dynamin, cholesterol, and a low pH intracellular environment (26). Following internalization, SADS-CoV viral particles depend on Rab5 for transport into early endosome, rely on Rab7 for transport from early endosome to late endosome or lysosome, and require Rab9 for transport from late endosome to Golgi apparatus (26). Finally, the viral genome is released to the cytoplasm.

**Figure 4:**
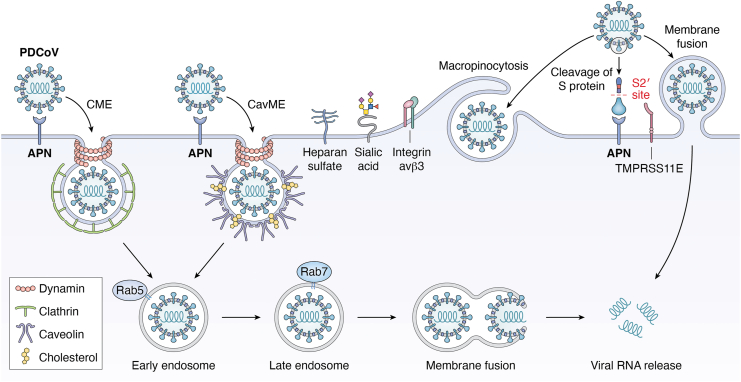
**Schematic diagram of PDCoV entry pathways.** Multiple cell-surface molecules take part in PDCoV entry, including APN, heparan sulfate, sialic acid, integrin avβ3, and TMPRSS11E (summarized in Tables 2 and 3). After binding to target cells, PDCoV virions are internalized through clathrin-mediated endocytosis (CME) and caveolae-mediated endocytosis (CavME) in a dynamin- and cholesterol-dependent manner. Trafficking of PDCoV viral particles from early endosome to late endosome requires the small GTPases Rab5 and Rab7. Then, membrane fusion takes place before viral RNA is released to the cytoplasm. Besides, PDCoV virions can enter target cells *via* macropinocytosis and membrane fusion at the cell surface through TMPRSS11E-mediated cleavage of S protein. APN, aminopeptidase N; PDCoV, porcine deltacoronavirus; TMPRSS, transmembrane protease serine.

PDCoV also utilizes endocytic and direct fusion routes to enter target cells, such as porcine ST and IPI-2I cells as well as human HuH7 cells (30, 31, 32, 33) (Fig. 4, which summarizes the pathways, receptors, cofactors, and other host factors used by PDCoV for cellular entry). Entry of PDCoV into ST cells is through CME and CavME, while entry into IPI-2I cells relies on CME and macropinocytosis, although entry into both cell types is dynamin- and low pH-dependent (30, 31, 32, 34, 35). However, PDCoV utilizes CavME, but not CME and macropinocytosis, to enter porcine kidney LLC-PK1 and PK15 cells (31, 36, 37). Interestingly, the pseudotyped viruses carrying PDCoV S protein can enter chicken embryo fibroblasts DF-1 cells, which is *via* CME in a dynamin 2-, Rab5-, Rab7-, and low pH-dependent manner (38). Taken together, these findings demonstrate that the routes of PDCoV entry are largely dependent on cell types.

Although the studies that revealed the entry pathways of porcine enteric coronaviruses were carried out in diverse cell types (Table 1), comparisons of their entry pathways based on currently available research findings could also draw several important points: (1) all porcine enteric coronaviruses TGEV, PEDV, SADS-CoV, and PDCoV can enter target cells through both CME and CavME; (2) SADS-CoV and PDCoV can enter target cells through macropinocytosis, although clarification studies are further needed for TGEV and PEDV; (3) PEDV, SADS-CoV, and PDCoV can enter target cells through direct fusion at the plasma membrane, although clarification studies are further needed for TGEV. Because swine enteric coronaviruses have potential risks of cross-species transmission, much work is warranted to define the detailed entry routes in every single susceptible cell type such that to find out the new and effective antiviral therapeutic measures to tackle the risks of interspecies transmission.

## Receptors, coreceptors, and cofactors

To get entry into target cells successfully, coronaviruses must first engage with key molecules on the cell surface, which are typically known as receptors or coreceptors. A receptor is an initial and specific binding site for the virus on the cell surface, which mediates initial virus attachment and guides the virus to the specific entry pathways. A receptor can be categorized into functional or nonfunctional one, with the former directly mediating viral entry into the cytoplasm of the cell, and the latter acting as an attachment receptor to concentrate the virus on the cell surface but failing to directly trigger viral entry. A coreceptor is a secondary, accessory binding site after virus engagement with primary receptors, which works in conjunction with primary receptors to facilitate viral entry and infection. With the assistance of certain cofactors, including furin, trypsin, transmembrane serine proteases, and cathepsins, S protein is cleaved to activate the fusion between viral and cellular membranes to accomplish the entry process. The receptors, coreceptors, and cofactors that are involved in the entry of TGEV, PEDV, SADS-CoV, and PDCoV into host cells are summarized in Table 2.

**Table 2:** The receptors, coreceptors, and cofactors involved in the cellular entry of TGEV, PEDV, SADS-CoV, and PDCoV

Virus	Molecule	Category	Functional annotation	Cell type	References
**TGEV**	APN	Functional receptor	The primary receptor, binding S1 subunit and mediating virus internalization by CME and CavME	ST, PD5, Thy, MDCK expressing pAPN, NIH3T3 and BHK21 expressing fAPN, BHK21 expressing dAPN, red fox APN, giant panda APN, cat APN, bAPN, hsAPN, pAPN and acAPN	(14, 54, 55, 56)
	TfR1	Functional receptor	A potential alternative receptor, binding S1 subunit and mediating virus entry^a^	ST, IPEC-J2, Caco-2 expressing porcine TfR1	(78)
	HSPA8	Coreceptor	Binds M protein, mediating virus internalization by CME	PK15	(81)
	EGFR	Coreceptor	Binds S1 subunit, mediating virus entry^a^	IPEC-J2	(19, 87)
	Sialic acid	Attachment receptor	Binds S protein, mediating virus attachment	LLC-PK1, ST	(123, 124)
PEDV	APN	Functional receptor	Binds S1 subunit, mediating virus internalization by CME and CavME, but not the major receptor	PK15, ST, porcine enterocytes, Vero, HuH7, MDCK expressing pAPN or hAPN	(48, 49, 50, 54)
	TfR1	Functional receptor	A potential alternative receptor, binding S1 subunit and mediating virus internalization by CME	IPEC-J2, Caco-2 and HEK293T expressing porcine TfR1	(77)
	HSPA5	Functional receptor	A potential alternative receptor, binding S protein and mediating virus attachment, internalization, and trafficking by the endo-lysosomal pathway	Vero, LLC-PK1	(82)
	DPP4	Coreceptor	Binds S1 subunit, mediating virus entry^a^	IPEC-J2	(94)
	FSTL1	Attachment receptor	Binds N and S2 proteins, mediating virus attachment	LLC-PK1	(99)
	TLR4	Attachment receptor	Binds N, S1, and S2 proteins, mediating virus attachment	LLC-PK1	(99)
	IFITMs	Coreceptor	IFITM1/2/3 bind S1 subunit, mediating virus entry^a^	HuH7, LLC-PK1	(108)
	RNASEK	Coreceptor	Binds S2, E, and M proteins, mediating virus internalization by CME	LLC-PK1	(112)
	Heparan sulfate	Attachment receptor	Mediates virus attachment, but which viral proteins bound have not been determined	Vero	(117)
	Sialic acid	Attachment receptor	Both α2,3- and α2,6-linked sialic acids bind S1 subunit, promoting virus attachment	LLC-PK1, Vero	(50, 126, 127)
	ST3GAL4^b^	/	Mediates virus attachment	LLC-PK1	(127)
	ST6GAL1^b^	/	Mediates virus attachment	LLC-PK1	(127)
	SLC35A1^b^	/	Mediates virus attachment by regulating ADAM17-mediated removal of pAPN ectodomain	LLC-PK1, Vero E6	(127, 130)
	DC-SIGN	Attachment receptor	Mediating virus entry^a^	BHK21 and NIH3T3 expressing hDC-SIGN or pDC-SIGN	(135)
	Siglec-15	Attachment receptor	Binds S1 and M proteins, mediating virus attachment	LLC-PK1, HEK293T, L929	(136)
	Trypsin	Cofactor	Cleaves S protein upstream of the fusion peptide	Vero, PK15, HuH7, IPI-2I	(22, 24, 126, 143, 144)
	TMPRSS	Cofactor	TMPRSS2/13 cleave S protein	Vero	(25)
	Cathepsins	Cofactor	Cathepsin B/L cleave S protein	PK15, HuH7, IPI-2I	(24)
SADS-CoV	Heparan sulfate	Attachment receptor	Mediates virus attachment, but which viral proteins bound have not been determined	Vero	(27)
	Sialic acid	Attachment receptor	Binds S1A subunit, mediating virus attachment	Vero, HuH7	(27)
	Furin	Cofactor	Cleaves S protein at the S1/S2 site	HEK293T, Vero	(27, 138)
	Trypsin	Cofactor	Cleaves S protein	Marc-145, Cos-7, BSC-40, Vero, ST, PK15, LLC-PK1, BHK21, L929, RlKi, PaKi, A549, HeLa, Hep2, RD, LLC-MK2, and Mv.1.Lu	(45, 146, 147)
	TMPRSS	Cofactor	TMPRSS2/13 cleave S protein	Vero, HuH7, HuH7.5.1, HeLa	(27, 28, 29)
	Cathepsins	Cofactor	Cathepsin B/L cleave S protein	Vero, HuH7	(146)
PDCoV	APN	Functional receptor	The primary receptor, binding S1 subunit and mediating virus internalization and membrane fusion	PK15, ST, IPI-2I, HuH7, Vero, BHK21 expressing dAPN, pAPN, acAPN, hAPN, rmAPN, mAPN, rAPN and chAPN, HeLa expressing pAPN, hAPN, gAPN, and fAPN, HEK293 and HEK293T expressing pAPN	(36, 41, 42, 43, 54, 56, 60)
	Heparan sulfate	Attachment receptor	Binds S1 subunit, mediating virus attachment	LLC-PK1, IPI-2I	(116)
	GLCE^c^	/	Binds S1 subunit, promoting attachment and internalization	LLC-PK1	(118)
	Sialic acid	Attachment receptor	Mediates virus attachment, dependent on trypsin	LLC-PK1, ST	(128)
	SLC35A1^b^	/	Mediates virus attachment by regulating the abundance of sialic acid	LLC-PK1, IPI-FX	(129)
	Trypsin	Cofactor	Cleaves S protein	LLC-PK1, IPI-2I, ST, PK15, IPAM	(30, 35, 145)
	TMPRSS	Cofactor	TMPRSS11E cleaves S protein	HuH7	(33)
	Cathepsins	Cofactor	Cathepsin B/L cleave S protein	ST, IPI-2I, HuH7	(30, 33)

A549, human lung adenocarcinoma cells; acAPN, Arabian camel APN; ADAM17, metalloprotease protein 17; BHK21, baby hamster kidney cells; Caco-2, human colorectal adenocarcinoma cells; chAPN, chicken APN; Cos-7, an African green monkey kidney fibroblast-like cell line with the expression of SV40T antigen; BSC40, an African green monkey kidney cell line adapted to growth at 40 °C; DC-SIGN, dendritic cell-specific intercellular adhesion molecule-3-grabbing nonintegrin; fAPN, feline APN; gAPN, galline APN; hDC-SIGN, human DC-SIGN; HEK293T, a derivative of the parental human embryonic kidney cells (HEK293) with the expression of SV40T antigen; HeLa, human cervical carcinoma cells; Hep2, human carcinoma cells derived *via* HeLa cell contamination; hsAPN, horse APN; HSPA5, heat shock protein family A member 5; HSPA8, heat shock protein family A member 8; HuH7, human hepatoma cells; HuH7.5.1, a subclone of the parent HuH7 cells; IFITM1/2/3, interferon-induced transmembrane protein 1, 2, and 3; IPAM, swine pulmonary alveolar macrophages; IPEC-J2, intestinal porcine epithelial cells-jejunum 2; IPI-2I, porcine ileum epithelial cells; IPI-FX, a derivative of the parental IPI-2I cells; L929, a mouse fibroblast cell line, NCTC clone 929 of strain L; LLC-MK2, *Macaca mulatta* kidney cells; LLC-PK1, porcine kidney cells; mAPN, mouse APN; Marc-145, meat animal research center-145, an African green monkey kidney cell line; MDCK, Madin-Darby canine kidney cells; Mv.1.Lu, *Mustla vison* lung cells; NIH3T3, mouse embryonic fibroblasts; PaKi, *Pteropus alecto* kidney cells; PD5, Philips-Duphar 5, porcine kidney cells; pDC-SIGN, porcine DC-SIGN; PK15, porcine kidney cells; rAPN, rat APN; RD, human rhabdomyosarcoma cells; RlKi, *Rousettus leschenaultia* kidney cells; rmAPN, Rhesus macaque APN; Siglec-15, sialic acid-binding immunoglobulin-type lectin 15; SLC35A1, solute carrier family 35 member A1 (SLC35A1); ST3GAL4, ST3 beta-galactoside alpha-2,3-sialyltransferase 4; ST6GAL1, ST6 beta-galactoside alpha-2,6-sialyltransferase 1; Thy, porcine thyroid cells; TMPRSS11E, transmembrane protease serine 11E; TMPRSS2/13, transmembrane protease serine 2 and 13, TMPRSS13 also known as mosaic serine protease large-form (MSPL); Vero, African green monkey kidney cells.

a The functional annotation involving “entry” indicates that a specific stage of virus attachment, internalization, or membrane fusion was not determined in the indicated literatures.

b Because the three molecules ST3GAL4, ST6GAL1, and SLC35A1 are essential for the synthesis of sialic acid, they are discussed in the paragraph of “Sialic acid” under the section of “The attachment receptors heparan sulfate, sialic acid, and lectin receptors” and thus not categorized here.

c Because GLCE is a key enzyme in HS synthesis, it is discussed in the paragraph of “Heparan sulfate” under the section of “The attachment receptors heparan sulfate, sialic acid, and lectin receptors” and thus not categorized here.

## Aminopeptidase N

Aminopeptidase N (APN), also known as cluster of differentiation 13 (CD13), a multifunctional zinc metalloproteinase ubiquitously expressed nearly in all types of cells, plays pivotal roles in various physiological and pathological processes, including cell-cell adhesion, sperm motility, angiogenesis, immune modulation, inflammation, carcinogenesis, and viral infections (39, 40). Early studies found that pAPN is the major receptor for TGEV (14) (Fig. 1), subsequent studies revealed that pAPN also functions as the major receptor for PDCoV (36, 41, 42, 43) (Fig. 4), whereas SADS-CoV does not use pAPN for cellular entry (44, 45). Consistent with the central role for pAPN in TGEV and PDCoV entry, ribosomal protein SA (RPSA, also known as laminin receptor 1), the known receptor for dengue virus (46), that promotes pAPN expression demonstrates a proviral effect on TGEV and PDCoV replication, although its role in TGEV and PDCoV entry remains to be determined (47). Whether PEDV utilizes pAPN as a receptor to enter cells has been controversial (48, 49, 50, 51, 52, 53), which is largely due to the limitations of traditional analytical methods that fail to capture the detailed dynamic molecular events during viral entry. Until recently, a research group resolved this controversy through single-virus tracking technology, demonstrating that pAPN mediates PEDV internalization (54) (Fig. 2). However, the pAPN-mediated internalization (35%) for PEDV is less efficient than TGEV (80%) and PDCoV (75%), with an approximately 60 s slower of internalization speed (54). Overall, these findings clearly demonstrate that pAPN serves as an entry receptor for TGEV, PEDV, and PDCoV.

Porcine enteric coronaviruses exhibit tremendous potential of interspecies transmission, as exemplified by the observations that these viruses have the capability to infect various cell types from different species including humans. It is plausible that the broad receptor usage is related to cross-species transmission of swine enteric coronaviruses. In support of this, TGEV has been shown to recognize APN orthologs from multiple animal species, including Carnivora (dog, cat, red fox, and giant panda), Artiodactyla (pig, bovine, and Arabian camel), and Perissodactyla (horse) (55, 56), with the dog APN (dAPN) displaying the highest binding activity and the residues Q746 and T749 being identified to be critical for binding to the RBD region of TGEV S glycoprotein (56). Likewise, PDCoV has the capability to bind APN orthologs from multiple species, including Primates (human and Rhesus macaque), Carnivora (dog and cat), Rodentia (mouse and rat), Artiodactyla (pig and Arabian camel), and Galliformes (chicken) (42, 56, 57). Notably, dAPN also displayed the highest binding activity to the RBD region of PDCoV S glycoprotein, and the residue R325 of dAPN was identified to be crucial for the binding (56). Taken together, these findings provide the theoretical basis for a better understanding of the potential interspecies transmission of porcine enteric coronaviruses.

It is noteworthy that although structural resolution revealed that PDCoV RBD binds to conserved regions on human APN (hAPN) and pAPN and exhibits a higher binding affinity to hAPN than that to pAPN (58), PDCoV is rarely reported to infect humans. To date, only one recent study has reported the infection of PDCoV in three Haitian children with acute undifferentiated febrile illness (59). It is plausible that this is closely related to the dynamic differences of hAPN and pAPN usage during PDCoV entry. In support of this, a recent study utilized single-virus tracking to visualize and dissect the dynamics of APN-mediated PDCoV entry and demonstrated that PDCoV recruited clathrin and caveolin-1 faster and internalized into host cells more quickly through CME or CavME following binding to low-affinity pAPN, as compared to high-affinity hAPN (60). Furthermore, low-affinity pAPN mainly induced cell surface fusion, whereas high-affinity hAPN primarily triggered endosomal fusion, which may reflect the evolution strategy of PDCoV for an optimal balance between immune evasion and rapid infection (60). Cumulatively, these findings demonstrate the capability of cross-species transmission of PDCoV and highlight the necessity of active surveillance for PDCoV infection.

Porcine enteric coronaviruses primarily target intestinal epithelial cells and cause severe pathological lesions in the intestines (3). Given that pAPN plays a dominant role in TGEV and PDCoV entry as discussed above, it is reasonable to assume that pAPN may determine their tropisms of infection in intestines *in vivo*. In support of this, a recent study established a PDCoV infection model of crypt-derived enteroids from different intestinal segments, and demonstrated that the expression of pAPN determined the intestinal segmental tropism of PDCoV (61). However, whether pAPN also plays a critically important role in the TGEV tropism of intestinal segments remains to be determined.

## Transferrin receptor 1

Transferrin receptor 1 (TfR1), also known as CD71, is a dimeric type II transmembrane glycoprotein that binds iron-loaded transferrin and promotes iron transport into cells through the CME pathway, thereby playing a crucial role in maintaining iron homeostasis (62). Infections of newborn piglets by swine enteric coronaviruses typically result in more severe diseases than those in older weaned and adult pigs (63, 64, 65, 66), but the reason for this remains elusive. Because neonatal piglets grow fast and fail to store iron timely, they are commonly deficient in iron. It is notable that the expression of TfR1, an entry factor involved in the infection of numerous viruses (67, 68, 69, 70, 71, 72, 73, 74), is tightly modulated by iron concentrations within the cells and iron deficiency results in a significantly increased level of TfR1 (75). Thus, it is not surprising that TfR1 plays a central role in maintaining the homeostasis of the intestines (76). Therefore, it is plausible that the high expression of TfR1 in newborn piglets is closely related to their high susceptibility to swine enteric coronaviruses. In support of this, high levels of TfR1 on the surface of intestinal epithelial cells in iron deficient newborn piglets have been shown to promote PEDV infection (77). Mechanistically, TfR1 utilizes its extracellular region to bind S1 subunit following PEDV infection, then clusters and recruits Src kinase to activate its phosphorylation at tyrosine 20, then induces the formation of clathrin coated endocytic vesicles and facilitates TfR1 internalization, thus increasing PEDV entry (77) (Fig. 2). Together, these findings strongly suggest that TfR1 functions as a receptor to mediate PEDV entry and that the higher levels of TfR1 in the apical tissue of the intestinal villi triggered by iron deficiency may determine the increased susceptibility of newborn piglets to PEDV, as compared to older weaned and adult pigs. Consistent with this, the involvement of TfR1 in TGEV entry has also been confirmed (78) (Fig. 1). Nevertheless, whether TfR1 is involved in the cellular entry of SADS-CoV and PDCoV has not been determined.

## Heat shock proteins

Heat shock proteins (HSPs) are a large group of molecular chaperones ubiquitously expressed in prokaryotic and eukaryotic cells. In addition to the classical chaperone functions of protein folding, refolding metastable proteins, and dissociating protein aggregates, HSPs also performs multiple important biological functions, including cell cycle, apoptosis, and signaling transduction (79, 80). Based on the molecular weights, HSPs are classified into HSP90, HSP70, HSP60, HSP40, and small HSPs families (79). Recently, two HSP70 family members, HSP family A member 8 (HSPA8, also known as heat shock cognate 71 KDa protein, HSC70) and HSPA5 (also known as glucose-regulated protein 78 kDa, GRP78, or binding-immunoglobulin protein, BiP), have been found to be involved in the infection of porcine enteric coronaviruses (81, 82). HSPA8 utilizes its substrate-binding domain to interact with TGEV M protein and facilitates virus internalization through the CME pathway (81) (Fig. 1). In contrast, HSPA5 binds PEDV S protein by its nucleotide-binding structural domain and promotes both attachment and internalization of PEDV (82) (Fig. 2). Moreover, HSPA5 is internalized together with PEDV into cells through endocytosis and increases the trafficking of PEDV *via* the endolysosomal pathway (82). However, the functional roles of HSPA8 and HSPA5 in the cellular entry of other porcine enteric coronaviruses are still unexplored. Also, it will be very interesting to test whether other HSPs are involved in the cellular entry of swine enteric coronaviruses because of the large numbers and versatile functions of HSP family members.

## Epidermal growth factor receptor

Epidermal growth factor receptor (EGFR) is a multifunctional transmembrane protein that is activated by binding to specific ligands, such as epidermal growth factor and other similar growth factors, which triggers multiple downstream signaling pathways that modulate cell proliferation, differentiation, and survival (83). Numerous studies have revealed that EGFR is a multifaceted player in viral life cycle, with the ability to regulate viral entry, viral replication, as well as immune evasion (84, 85, 86). In agreement with this, EGFR has been shown to promote TGEV entry by interacting with TGEV S1 protein *via* its extracellular receptor binding domain 1 (19, 87) (Fig. 1). Notably, EGFR and APN can synergistically facilitate the entry of TGEV into porcine intestinal epithelial cells and activate phosphatidylinositol 3-kinase (PI3K)-AKT and mitogen-activated protein kinase (MEK)/extracellular signal-regulated kinase (ERK) signaling pathways (19). Besides, it is noteworthy that while TGEV utilizes EGFR as an accessory receptor for entry, the role of EGFR in PEDV infection seems to be very different. PEDV is able to activate EGFR signaling to dampen antiviral activity of the type I interferon response, thus promoting PEDV infection (88). However, whether EGFR also plays a proviral role in the cellular entry of PEDV has not been elucidated.

## Dipeptidyl peptidase 4

Dipeptidyl peptidase 4 (DPP4), also known as CD26 or adenosine deaminase complexing protein 2 (ADCP2), is a serine exopeptidase that cleaves dipeptides from the N terminus of polypeptides. It is a type II transmembrane glycoprotein extensively expressed on the surface of numerous cell types and plays a key role in multiple important physiological processes, including glucose metabolism, immune regulation, and signal transduction (89, 90). DPP4 is well known as the entry receptor for several bat and human coronaviruses, including Tylonycteris-bat-CoV-HKU4, human coronavirus-Erasmus Medical Center (hCoV-EMC), and Middle East respiratory syndrome coronavirus (MERS-CoV) (91, 92, 93). Furthermore, it has recently been shown that DPP4 may also function as an accessory receptor for PEDV entry (94) (Fig. 2). However, the entry of SADS-CoV into target cells does not require DPP4 (44, 45, 95).

## Follistatin-like 1 and toll-like receptor 4

Follistatin-like 1 (FSTL1) is a secreted extracellular matrix glycoprotein that participates in multiple important physiological and pathological processes, including angiogenesis, cell proliferation and differentiation, immune regulation, inflammation, and tumorigenesis (96, 97, 98). Studies on the role of FSTL1 in viral infection are rarely reported. There is only one recent study showing that FSTL1 promotes PEDV adsorption to host cells by interacting with PEDV N and S2 proteins (99) (Fig. 2). Mechanistic studies demonstrated that FSTL1 interacts with toll-like receptor 4 (TLR4), a well-known pattern recognition receptor involved in the recognition of numerous viruses, bacteria, and fungi (100), and enhances TLR4 recognition of PEDV N, S1, and S2 proteins, thus facilitating virus attachment to target cells (99). However, whether FSTL1 and TLR4 also function as mediators for the attachment of TGEV, SADS-CoV, and PDCoV to target cells remains unknown.

## Interferon-induced transmembrane proteins

Interferon-induced transmembrane proteins (IFITMs) are ubiquitously expressed and can be induced by type I, type II, and type III interferons. They are well recognized as central players in innate immunity, adaptive immunity, and cancer development (101, 102). The roles of IFITM proteins in viral infections are complex, with either antiviral or proviral effects in a context-dependent fashion. IFITM proteins are thought to act as broad-spectrum antiviral factors that restrict the replication of viruses from various families, including flaviviruses (dengue virus, West Nile virus, hepatitis C virus, Zika virus, and yellow fever virus), rhabdoviruses (vesicular stomatitis virus), filoviruses (Ebola virus and Marburg virus), bunyaviruses (Rift Valley fever virus and La Crosse virus), orthomyxoviruses (influenza A virus), paramyxoviruses (parainfluenza virus, metapneumovirus, and respiratory syncytial virus), alphaviruses (chikungunya virus, Sindbis virus, and Semliki Forest virus), lentiviruses (human and simian immunodeficiency viruses), poxviruses (vaccinia virus and cowpox virus), and coronaviruses (human coronavirus 229E, MERS-CoV, severe acute respiratory syndrome coronavirus (SARS-CoV), SARS-CoV-2, and TGEV) (101, 102, 103). They primarily inhibit the fusion between viral and cellular membranes at the viral entry stage, but can also act at later stages of viral life cycle (101, 102). In contrast, studies on the proviral roles of IFITM proteins are relatively less. IFITM proteins promote the cellular entry of human coronavirus OC43, Nipah virus, human cytomegalovirus, hepatitis B virus, and hepatitis D virus (104, 105, 106, 107). Interestingly, while IFITM1-3 interact with PEDV S1 subunit, only IFITM2/3 (but not IFITM1) act as entry factors for PEDV infection in human cells, and only IFITM1 (but not IFITM2/3) promotes PEDV entry into porcine cells and porcine small intestinal organoids (108) (Fig. 2). Taken together, these findings suggest that IFITM proteins may exert either antiviral or proviral activities against coronaviruses. The reason for this is still unknown, but it is likely to be associated with the differences of cell types and virus strains used.

## Ribonuclease kappa

Ribonuclease kappa (RNASEK) is a highly conserved transmembrane protein that is an important component of vacuolar ATPase proton pump involved in regulating pH of intracellular compartments. Apart from acting as a positive regulator of autophagosome degradation (109), RNASEK is required for both clathrin-mediated and clathrin-independent endocytic uptake of a large number of viruses, including human rhinoviruses, dengue virus, influenza virus, West Nile virus, Sindbis virus, and Rift Valley fever virus (110, 111). Because PEDV enters host cells through the endocytic pathway (Table 1), it is not surprising that RNASEK also plays a crucial role in the endocytic uptake of PEDV (112). RNASEK interacts with PEDV S2, E, and M proteins, and promotes the internalization of PEDV into target cells by CME (112) (Fig. 2). It is likely that RNASEK is also involved in the internalization of TGEV, SADS-CoV, and PDCoV, given that these viruses also utilize the endocytic pathway to enter cells (Table 1). However, the related evidence is lacking, and it needs to be experimentally validated.

## The attachment receptors heparan sulfate, sialic acid, and lectin receptors

Heparan sulfate (HS) is a highly sulfated polysaccharide abundantly expressed on the cell surfaces and in the extracellular matrix of animal tissues. Numerous viruses employ HS as their attachment receptors to bind cells to initiate infection, including porcine enteric coronaviruses (27, 113, 114, 115, 116, 117). Except for TGEV, the other three swine enteric coronaviruses PEDV, SADS-CoV, and PDCoV utilize HS as the attachment receptors (27, 116, 117) (Figure 2, Figure 3, Figure 4). Consistent with a critical role of HS in PDCoV entry (116), D-glucuronyl C5-epimerase (GLCE), a key enzyme in the synthesis of HS, promotes PDCoV attachment and internalization by interacting with PDCoV S1 protein (118). Moreover, GLCE can promote the direct binding of PDCoV S1 protein to pAPN, and act synergistically with pAPN to enhance PDCoV infection (118). However, whether GLCE is also involved in regulating the cellular entry of PEDV and SADS-CoV remains to be determined.

Sialic acid is a type of acid sugar that is widely distributed at the terminal end of glycoproteins and glycolipids on the surface of various human and animal cells, and plays pivotal roles in cellular communication and function (119, 120). A wide variety of viruses utilize sialic acids as attachment receptors for cellular entry (121, 122). Not surprisingly, porcine enteric coronaviruses also use sialic acid to attach to target cells to initiate infection (27, 50, 123, 124, 125, 126, 127, 128) (Figure 1, Figure 2, Figure 3, Figure 4). TGEV can recognize both N-acetylneuraminic acid (Neu5Ac) and N-glycolylneuraminic acid (Neu5Gc), but PEDV only recognizes Neu5Ac (50, 123). It is notable that although the sialic acid binding activity of TGEV S protein is dispensable for infection of cultured cells, it is likely to be required for infection of intestinal cells (124, 125). Consistent with the critical role for sialic acid in the cellular entry of swine enteric coronaviruses (27, 50, 123, 124, 125, 126, 127, 128), a recent genome-wide CRISPR-Cas9 screen revealed that the host factors involved in sialic acid synthesis, including ST3 beta-galactoside alpha-2,3-sialyltransferase 4 (ST3GAL4), ST6 beta-galactoside alpha-2,6-sialyltransferase 1 (ST6GAL1), and solute carrier family 35 member A1 (SLC35A1), are of vital importance for the attachment of PEDV to target cells (127). Furthermore, SLC35A1 plays a proviral role in the attachment of PDCoV by regulating the abundance of cell-surface sialic acid (129). Nevertheless, it seems that the role of SLC35A1 during PEDV entry is sialic acid-independent, and SLC35A1 promotes PEDV attachment by regulating metalloprotease protein 17 (ADAM17)-mediated removal of pAPN ectodomain (130). Taken together, these findings clearly demonstrate that sialic acid serves as a key attachment receptor for the cellular entry of TGEV, PEDV, SADS-CoV, and PDCoV, and reveal that the wide distribution of sialic acid on the cell surface may determine the broad host range of swine enteric coronaviruses.

Lectins are a large group of carbohydrate-binding cell-surface protein receptors, mainly composed of C-type lectins and sialic acid-binding immunoglobulin-type lectins (Siglecs) (131). Similar to other pathogen recognition receptors, they also play important roles in regulating host innate and adaptive immunity (131). Moreover, lectin receptors can mediate the attachment and binding of various viruses, bacteria, and fungi to their target hosts (132, 133, 134). Specifically, the expression of C-type lectin DC-SIGN (dendritic cell-specific intercellular adhesion molecule-3-grabbing nonintegrin) in refractory cells increases the susceptibility of PEDV, revealing its crucial role in mediating PEDV entry (135) (Fig. 2). As a conserved type I transmembrane protein of the Siglecs family, Siglec-15 has recently been found to promote PEDV entry by binding the S1 and M proteins (Fig. 2), and a home-made monoclonal antibody against Siglec-15 demonstrated a potent antiviral activity against PEDV infection *in vivo* (136). These findings indicate that Siglec-15 represents an attractive and promising target for therapeutic interventions against PEDV. Despite their multifunctional effects in various physiological processes, the studies on the potentiality of lectins as entry receptors for swine enteric coronaviruses remain in the infancy and worth further investigations in the future.

## The cofactors furin, trypsin, transmembrane serine proteases, and cathepsins

Coronaviruses initiate infection by binding their S proteins to the cell-surface receptors. Activation of the fusion activity of S proteins requires proteolytic cleavage at two recognition sites by host proteases (8). The first site is located at the S1/S2 boundary, which results in conformational changes of S2 subunit to place it in a prefusion state. The second site is at S2′, located near or just upstream of the fusion peptide, which drives the fusion between viral and cellular membranes and thus allows the release of viral RNA genome to the cytoplasm. Cleavage at the two sites (S1/S2 and S2′) is typically mediated by a wide range of proteases, including proprotein convertase furin, trypsin or trypsin-like proteases, transmembrane protease serine (TMPRSS), and cathepsins (137). Although coronaviruses utilize this general strategy to activate S protein, the cleavage sites and proteases used differ dependent on the virus.

For porcine enteric coronaviruses, furin cleavage sites are only found in SADS-CoV and PDCoV S proteins (27, 33, 138), but not the counterparts of TGEV and PEDV (139, 140, 141, 142). Although PEDV S protein does not contain an active furin cleavage site, a potential cleavage site containing a conserved arginine just upstream of the fusion peptide can be cleaved by trypsin (143). This explains the reason for the supplementation of trypsin when propagating WT PEDV in cell culture (22, 24, 143, 144). Notably, PDCoV S protein is not cleaved by furin although it contains several potential furin cleavage sites (33). Consistent with this, furin plays a critical role in facilitating PDCoV maturation and release, rather than at the entry stage (33). Interestingly, exogenous addition of trypsin can increase PDCoV infection in porcine but not human cells (30, 33, 35, 145). For SADS-CoV S protein, the furin-mediated cleavage occurs near the S1/S2 site (27, 138), and trypsin is important for infection of many of its susceptible cells (45, 146, 147).

The TMPRSS family of proteins is type II transmembrane proteins that have serine protease activities, with 18 members identified so far. Among them, transmembrane protease serine 2 and 13 (TMPRSS2/13) are capable of cleaving PEDV and SADS-CoV S proteins, inducing membrane fusion at the cell surface and thus promoting viral entry in susceptible cells (25, 27, 28, 29) (Figs. 2 and 3). However, cleavage of PDCoV S protein and induction of subsequent membrane fusion is dependent on transmembrane protease serine 11E (TMPRSS11E) (33) (Fig. 4). Cathepsins are a family of proteases primarily found in endosome and lysosome that digest proteins in the acidic environment. Because PEDV, SADS-CoV, and PDCoV can use the endocytic pathway to enter host cells (Table 1), it is not surprising that the lysosomal proteases cathepsin B and L cleave their S proteins to activate membrane fusion in the endosome (24, 30, 33, 146).

## Other host factors used by porcine enteric coronaviruses for cell entry

During virus entry, the host factors may function at different stages or through different mechanisms. In this section, we discuss the host factors that are not appropriate to be categorized as receptors, coreceptors, or cofactors, and thus designate them as “other host factors”. For example, although some host factors are transmembrane proteins, their interactions with S protein have not been identified; or while they are not transmembrane proteins, they utilize various mechanisms to promote the cellular entry of porcine enteric coronaviruses. These “other host factors” are summarized in Table 3.

**Table 3:** The other host factors involved in the cellular entry of TGEV, PEDV, SADS-CoV, and PDCoV

Molecule	Virus	Functional annotation	Cell type	References
DYRK1A	TGEV	Promotes virus attachment and internalization by enhancing pAPN gene expression	PK15	(150)
DR5	PEDV	Mediates virus attachment, but whether directly binding to PEDV has not been determined	Vero	(153)
eIF4E	PEDV	Promotes virus attachment by upregulating membrane-residential host proteins TSPAN3, CD63, and ITGB2	Vero	(155)
Integrin αvβ3	PEDV	Mediates virus internalization, but whether directly binding to PEDV has not been determined	Vero E6, IEC	(163)
	PDCoV	Mediates virus attachment and internalization, but whether directly binding to PDCoV has not been determined	ST, LLC-PK1	(35)
TSG101	SADS-CoV	Promotes virus transport to lysosome	Vero, primary porcine jejunal cells	(166)
ALIX	PEDV	Promotes virus transport to lysosome	Vero, primary porcine jejunal cells	(166)
	SADS-CoV	Promotes virus transport to lysosome	Vero, primary porcine jejunal cells	(166)

ALIX, ALG-2-interacting protein X, also known as programmed cell death 6 interacting protein (PDCD6IP); DYRK1A, dual-specificity tyrosine phosphorylation-regulated kinase 1A; IEC, porcine intestinal epithelial cells; ITGB2, integrin subunit beta 2; LLC-PK1, porcine kidney cells; PK15, porcine kidney cells; ST, swine testis cells; TSG101, tumor susceptibility gene 101; TSPAN3, tetraspanin 3; Vero E6, African green monkey kidney cells, clone E6; Vero, African green monkey kidney cells.

## Dual-specificity tyrosine phosphorylation-regulated kinase 1A

Dual-specificity tyrosine phosphorylation-regulated kinase 1A (DYRK1A) is a multifunctional protein kinase with both nuclear and cytoplasmic location that is known to regulate cell proliferation, apoptosis, and tumorigenesis (148). Similar to the critical role identified for SARS-CoV, SARS-CoV-2, and MERS-CoV entry (149), DYRK1A has also been demonstrated to promote the attachment and internalization of TGEV by elevating the expression of pAPN (Fig. 1), the major functional receptor for TGEV (14), as well as the endocytic-related genes (150). In addition to the impact on viral entry, DYRK1A can assist in the formation of double-membrane vesicles in a kinase-independent manner, thus facilitating TGEV replication (150). Notably, it seems that the proviral role of DYRK1A is conserved since the other RNA viruses, including PDCoV, mouse hepatitis virus, and porcine sapelovirus, also rely on it for efficient replication (150).

## Death receptor 5

Death receptor 5 (DR5), also known as tumor necrosis factor (TNF) receptor superfamily member 10B (TNFRSF10B) or TNF-related apoptosis-inducing ligand (TRAIL) receptor 2 (TRAILR2), is an important cell-surface receptor that mediates caspase 8 activation and apoptosis (151). Although DR5 exhibits both antiviral and proviral activities, its role in viral infection is typically associated with its apoptosis function (152, 153). For example, DR5 has been shown to function as a restriction factor to inhibit lytic replication of the human Kaposi sarcoma-associated herpesvirus (KSHV), which relies on DR5-induced apoptosis (152). In contrast, in the case of PEDV infection, DR5 expression is upregulated to promote viral replication by modulating caspase-8-dependent apoptosis (153). It is notable that DR5 has the capability to promote PEDV attachment (Fig. 2), which seems to be independent of its apoptosis function (153). However, whether DR5 directly binds PEDV proteins has not been determined. In addition, it will be very interesting to test whether DR5 is also involved in the cellular entry of other porcine enteric coronaviruses in an apoptosis-independent manner.

## Eukaryotic initiation factor 4E

Eukaryotic initiation factor 4E (eIF4E) is an integral component of the eukaryotic translation initiation factor 4F complex with both nuclear and cytoplasmic location, which facilitates efficient protein synthesis by recruiting ribosomes to the 5′-cap structure of mRNAs (154). The translation activity of eIF4E is generally dependent on its phosphorylation at serine residue. Interestingly, the phosphorylation of eIF4E can be induced at the early stage of PEDV infection through the ERK-mitogen-activated protein kinase (MAPK) interacting protein kinase (MNK) pathway (155). Phosphorylated eIF4E (p-eIF4E) elevates the expression of membrane-residential host factors tetraspanin 3 (TSPAN3), CD63, and integrin subunit beta 2 (ITGB2) at the translation level rather than at the transcription level to promote PEDV attachment (155) (Fig. 2). Moreover, TSPAN3, CD63, and ITGB2 facilitates the efficient entry of SARS-CoV and SARS-CoV-2 S protein pseudotyped viruses (155). Remarkably, p-eIF4E is also triggered at the early times following infection with diverse viruses, including TGEV, human coronavirus OC43, and herpes simplex virus type 1, implicating that p-eIF4E may also be important for mediating the cellular entry of these viruses. These findings are very interesting because they reveal for the first time the relationship between p-eIF4E stimulation and membrane-residential host factors in viral entry.

## Integrin αvβ3

Integrins are a large family of transmembrane glycoproteins that play important roles in regulating a wide range of biological functions, including cell adhesion, cell migration, cell proliferation, cell survival, and various signaling processes (156, 157, 158). There are 24 integrins identified so far, with each integrin heterodimer consisting of an α-subunit and a β-subunit, of which there are 18 and 8 variants, respectively (156, 157, 158). As one of the important integrins, integrin αvβ3 is well-known in the field of virology for its central role in mediating cellular entry of several viruses (159, 160, 161, 162). Thus, it is not surprising that integrin αvβ3 has also been reported to play an important role in the infection of swine enteric coronaviruses (35, 163). Although integrin αvβ3 does not affect PEDV attachment, it is able to promote PEDV internalization in synergistic with pAPN (163) (Fig. 2). In contrast, integrin αvβ3 facilitates both attachment and internalization of PDCoV (Fig. 4), and importantly, it may promote early entry of PDCoV by activating the focal adhesion kinase (FAK)-PI3K-AKT signaling pathway (35). Although these exciting findings indicate that integrin αvβ3 may promote cellular entry of both PEDV and PDCoV, more evidence, such as the identification of its interaction with S protein, remains to be determined.

## The ESCRT and related proteins

The endosomal sorting complexes required for transport (ESCRT) system is a cellular machinery comprised of several evolutionarily conserved multisubunit membrane remodeling complexes (ESCRT-0, ESCRT-I, ESCRT-II, and ESCRT-III) and the associated accessary proteins, which primarily assists in sorting and moving certain proteins inside cells. As a key subunit of ESCRT-I complex, tumor susceptibility gene 101 (TSG101) protein interacts with its accessory protein ALG-2-interacting protein X (ALIX), which contributes to the biogenesis of multivesicular bodies to transport ubiquitinated proteins for degradation in the lysosome (164, 165). Thus, TSG101 and ALIX play important roles in endosomal trafficking. Because porcine enteric coronaviruses can enter target cells through endocytic pathways (Table 1), it is likely that TSG101 and ALIX participate in their entry processes. Indeed, a recent study demonstrated that TSG101 and ALIX are required for cellular entry and replication of PEDV and SADS-CoV (166). Although ALIX is crucial for the entry of both PEDV and SADS-CoV, TSG101 is specifically important for SADS-CoV entry (166). Upon infection with PEDV and SADS-CoV, ALIX is recruited to the caveolin-1 rich site and then to the late endosome (Figs. 2 and 3), thereby promoting virus transport to lysosome (166). In contrast, TSG101 is recruited to macropinocytosis endocytosis site first, then to the late endosome, following SADS-CoV infection (166) (Fig. 3). Interestingly, both ALIX and TSG101 can be recruited to help PEDV and SADS-CoV form double membrane vesicles in the endoplasmic reticulum, thus promoting viral replication (166). Taken together, these findings reveal distinct requirements for ALIX and TSG101 in the endo-lysosomal transport processes of PEDV and SADS-CoV.

## Concluding remarks

Porcine enteric coronaviruses are not only serious veterinary concerns in the swine industry, but also potential threats to public health. Particularly, PEDV has been circulating in many countries in Asia, North America, and Europe, and PDCoV has also been demonstrated to infect Haitian children (2, 59). The past and recent fascinating work has led to identification of several key pathways and receptors for cellular entry of TGEV (Fig. 1), PEDV (Fig. 2), SADS-CoV (Fig. 3), and PDCoV (Fig. 4). These exciting findings have enabled us to obtain deep insights into the tropism, host range, cross-species transmission, pathogenesis, and intervening measures of these economically important and publicly concerned porcine viruses. For instance, the pigs deficient in APN, the major receptor for TGEV (14), displayed resistant to highly virulent TGEV challenge, and TGEV antigen was undetectable in target tissues of APN knockout pigs (167, 168). Furthermore, neutralizing antibodies directed against PDCoV S glycoprotein disrupted the binding of PDCoV to the entry receptor APN to neutralize virus infectivity (9, 10). Notably, a neutralizing antibody recognizing a conserved conformational epitope in PDCoV S1 protein exhibited strong therapeutic efficacies in PDCoV-infected piglets, with delayed onset of diarrhea symptoms, reduced severity of diarrhea, and decreased virus shedding (11). These findings reveal that the amino acid sequences recognized by these neutralizing antibodies may provide valuable and promising targets for developing novel epitope-based vaccines and antiviral agents.

Despite remarkable achievements of studying the entry mechanisms of porcine enteric coronaviruses, significant questions remain. For example, most of the work was not carried out in the enterocytes, which may not reflect the real scenes of virus infection in the intestines. Thus, it is mandatory to further find out the real functions of the reported entry pathways, receptors, coreceptors, cofactors, and other host entry factors in the intestinal cells or even in the intestinal organoids. Most of the identified receptors and entry factors are only verified in the infection of one type of virus, the potential shared functions of these molecules in the cellular entry of multiple porcine enteric coronaviruses remain to be identified. This is critically important because it is very valuable for finding out key targets for developing broad-spectrum antiviral therapeutical measures. Although several receptors have been identified for TGEV, PEDV, and PDCoV, whether other receptors for these viruses exist remain to be determined. Moreover, the specific functional receptors for SADS-CoV are completely unknown. Therefore, systematic identification of novel receptors or entry factors for these viruses through high-throughput CRISPR-Cas9, ORF, and RNAi screens will be an important focus of future research. In addition, development of single-virus tracking technology greatly advances the studies of virus entry, which contributes to dissecting the detailed dynamics of single virion entering cells. This technology will certainly continue to be applied in future studies to specifically identify the dynamic roles of various receptors and entry factors. Finally, with the development of various new technologies and an increasing number of researchers devoting themselves to studying virus entry, combined with the experiences accumulated during the study of SARS-CoV-2 entry, the investigation and understanding of the entry mechanisms of porcine enteric coronaviruses will be greatly expedited.

## Conflict of interest

The authors declare that they have no conflicts of interest with the contents of this article.
